# Hydrogels and nanogels: effectiveness in dermal applications

**DOI:** 10.3762/bjnano.16.90

**Published:** 2025-08-01

**Authors:** Jéssica da Cruz Ludwig, Diana Fortkamp Grigoletto, Daniele Fernanda Renzi, Wolf-Rainer Abraham, Daniel de Paula, Najeh Maissar Khalil

**Affiliations:** 1 Pharmacy Department, Applied Nanoestructured Systems Laboratory, Universidade Estadual do Centro-Oeste, Alameda Élio Antonio Dalla Vecchia, 838 - CEP 85040-167 - Bairro - Vila Carli, Guarapuava – PR, Brazil; 2 Helmholtz-Zentrum für Infektionsforschung, Inhoffenstrasse 7, 38124 Braunschweig, Germany

**Keywords:** cross-linking, drug delivery, formulation, nanogel, polymer

## Abstract

Drug delivery systems (DDSs) are an important tool for obtaining medicines with improved physicochemical properties, especially for drugs with stability, absorption, and biodistribution impairments. Among the DDSs, we can highlight hydrogels and nanogels, which are easy to obtain, show good biocompatibility, and have several applications in the design of drug carriers for dermal and ocular administration. In this review, we introduce a brief concept on hydrogels, underlining compounds such as chitosan and alginate, and methods used for their preparation. Nanogels, with their attractive features, such as high drug encapsulation and penetration enhancer embedding, are also addressed. Finally, the application of these systems in dermal pathophysiological processes through the incorporation of drugs for enhancing skin permeation brings out promising prospects for innovation which may arise in the drug delivery field.

## Introduction

The systematic study of gels began in the mid-1950s and 1960s [1–4]. From the beginning, hydrogels composed of cross-linked acrylic acid were designed to release drugs into the body [5]. 2-Hydroxyethyl methacrylate was one of the first compounds used to build hydrogels as drug delivery systems (DDSs) [6]. Over the past century, hydrogels have emerged as promising alternatives for various medical purposes, including skin [7], nasal [4], and eye [8] applications. The evolution of ocular contact lenses, for example, has benefited from the development of hydrogels [9–10].

Hydrogels can be described as cross-linked polymeric networks embedded in hydrophilic solvents, usually water, which can carry active materials and biomolecules [11–13]. After hydration in an aqueous environment, the hydrogel structure is created by hydrophilic groups or regions arranged in a polymeric network [14]. Hydrogels present interesting properties, comprising high biocompatibility and three-dimensional conformation. Their structural organization simulates the architecture of living tissues, allowing the flux of nutrients, oxygen, metabolites, and even whole cells. As a result, hydrogels can be molded into body parts, being successfully employed in regenerative tissue engineering [11–13,15].

Macroscopic hydrogel networks can be decoupled into microscale particles of any shape, called microgels. When gelatinous particles are configured in the submicrometric range, for example, with diameters in the order of nanometers, they are known as nanogels [16–17]. Nanogels can be obtained from synthetic and natural polymers and can absorb water up to a thousand times their weight, which represents 99.9% of their content [18–19].

Nanogels have a wide field of applicability that covers several areas of science. They have proved useful for oil extraction [20–21], drug monitoring in biological fluids [22], CO_2_ absorption from the atmosphere [23], dye removal from industrial effluents [24], cavity-prevention in oral products [25], insect repellent coating for textiles [26], lead detection in water reservoirs [27], production of vaccines [28], and prevention of the spontaneous combustion of coal [29].

The development of chemically functional materials on the nanoscale appears to be of fundamental importance when it comes to health applications. Nanogels are an excellent alternative for the manufacture of biomaterials due to their physical and chemical properties, which are like living tissues [30–31]. Beyond their application in tissue engineering, nanogels are promising materials as DDSs [32]. Nanogels have been successfully designed as drug carriers for oral [33–34], nasal [35], ocular [36], dermal [37], and intravenous [38] administrations.

As the largest organ in the body, the human skin has a critical role in the maintenance of vital functions: it provides protection to all internal organs, contributes to immune responses, and receives sensorial stimuli from the external environment [39]. The complexity of human skin makes it challenging to mimic. On the other hand, hydrogels and nanogels have arrived on the market as materials that resemble skin tissue, offering a hygroscopic and favorable environment for wound healing, for example. They can be combined with active ingredients that accelerate the primary goal of healing injured skin, helping to improve treatment effectiveness [40–42].

This paper reviews the preparation methods of hydrogels and nanogels from hydrophilic polymers of synthetic and natural origin with an emphasis on cross-linking reactions by physical and chemical methods. Additionally, recent advancements in the dermal application of hydrogels and nanogels as DDSs are particularly addressed.

## Review

### Preparation of hydrogels

**Figure 1:**
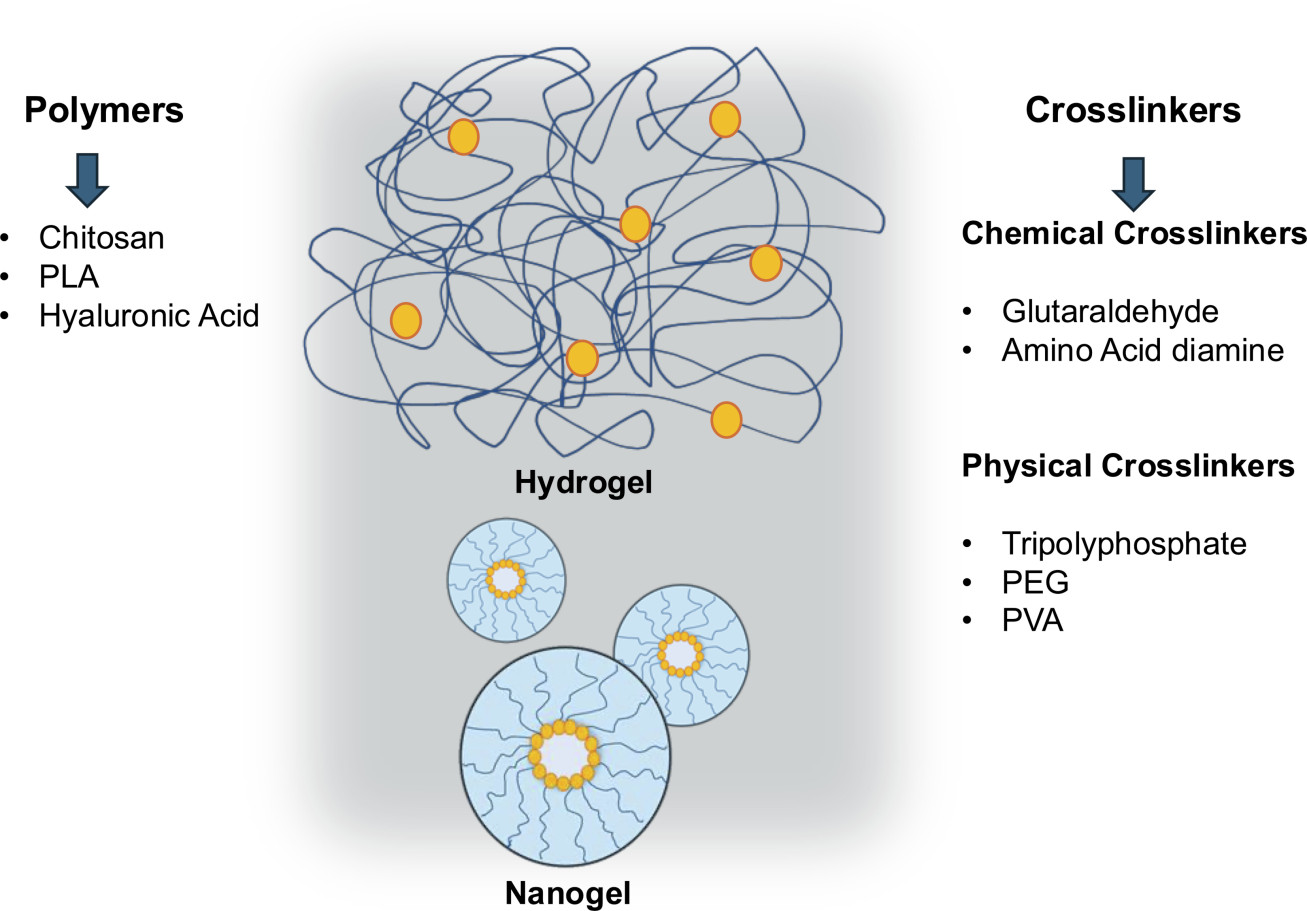
Structure of hydrogels and nanogels of cross-linked polymers. Cross-linking is a technique for forming hydrogels, a process in which polymer chains are joined through covalent and noncovalent bonds, forming three-dimensional networks. The process allows polymers to be cross-linked intra- or intermolecularly by chemical bonds or physical interaction.

Cross-linking, one of the main techniques for hydrogel formation, is the process of linking polymer chains by covalent or noncovalent bonds, forming tridimensional networks [43–44]. Polymers can be intra- or intermolecularly cross-linked by chemical bonding or physical interaction (Figure 1). Physical cross-linking is performed using interactions other than the covalent bond, such as hydrogen bonding or ionic interaction. Physical cross-links can be reversibly dissociated and recombined under specific stimuli such as heating/cooling [45–46]. Polymer chemical cross-linking can be performed by the formation of a network structure from monomers by polymerization or post-cross-linking of linear polymers with a cross-linking agent. Examples of chemical cross-linking methods are covalent bonding between polymer chains accomplished by irradiation, condensation reaction, radical polymerization, or chemical reactions [14,43,47].

Cross-linking agents or cross-linkers are symmetrical bifunctional compounds that contain two or more reactive ends capable of chemically attaching to specific functional groups, such as primary amines and sulfhydryl groups on proteins or other biomolecules. The reactive chemical group is the most important property of a cross-linker. The reactive group establishes the method and mechanism for polymer modification [43,45]. Harsh conditions such as low pH and high temperature may be required depending on the degree of reactivity between the polymer-cross-linker [47] and the desired characteristics of the hydrogel [14].

Various cross-linkers that cannot be categorized as conventional chemical cross-linkers have been utilized for the formation of hydrogels and nanogels [48–51]. Interpenetrating polymer networks (IPNs) are materials composed of two or more polymer networks that remain chemically cross-linked through physical and inseparable interweaving, favoring the homogeneous mixing of immiscible polymers. By adding a polymer network with more flexible properties to a gel previously composed of a more rigid and brittle network, a double network gel with an IPN structure is obtained. This approach significantly contributes to improving the mechanical properties and stability of the system [43,52].

Synthetic polymers, including acrylates [53], acrylamide [54], polyvinyl alcohol (PVA) [55], as well as natural polymers, notably chitosan [56–57] and alginates [58–59], have been tested in hydrogel formulations both individually and in combinations [60–62]. The variety of compounds that can be used in hydrogel formulations explains the large number of methodologies designed to obtain them.

In addition to optimizing the physicochemical methods used to prepare nanogels and hydrogels, the application of precision engineering principles has proven essential to enhance the therapeutic performance of these formulations [63–64]. In developing nanocarriers, pharmacokinetic and pharmacodynamic parameters – such as size, release kinetics, and biodistribution of the encapsulated drug – must be carefully defined to maximize the efficacy of the system [65]. Such considerations are essential to obtain stable formulations with a controlled release profile that can be topically and systemically applied [66]. Table 1 summarizes the composition, cross-linkers, and preparation methods of hydrogels related to dermal applications.

**Table 1:** Composition, cross-linkers, and preparation methods of dermal-related hydrogels.

Polymer^a^	Concentration range	Cross-linker	Preparation methods	Ref.

acrylamide	15.0–30.0%	*N,N'*-methylene-bis-acrylamide	UV photo-polymerization, free-radical polymerization	[54,67]
alginate	0.8–1.5%	CaCl_2_, glutaraldehyde	ionic gelation, chemical cross-linking	[59,68–72]
cellulose	0.1–4.0%	epichlorohydrin, poly(ethylene glycol) diglycidyl ether, CaCl_2_/ZnCl_2_	phase inversion in ethanol, chemical cross-linking ionic gelation	[51,73–74]
chitosan	1.0–5.0%	tetrakis (hydroxymethyl) phosphonium chloride, glutaraldehyde, β-glycerophosphate, tripolyphosphate, genipin, silver nitrate	low pH physical cross-linking (e.g. acetic acid, citric acid, lactic acid), freeze–thawing, ionic gelation, chemical cross-linking	[40,48–49,75–81]
CMC	2.0%	–	physical cross-linking	[82–83]
collagen	0.3–1.0%	1-ethyl-3-(3-dimethylamino- propyl)carbodiimide, riboflavin	chemical cross-linking, temperature-oriented cross-linking, UV photo-cross-linking	[84–86]
gelatin	5.0–10.0%	*N*-(3-dimethylaminopropyl)-*N*'- ethylcarbodiamide hydrochloride), *N*-hydroxysuccinimide	UV photo-cross-linking, chemical cross-linking	[87–88]
HEC	1.5%	poly(ethylene glycol) diacrylate	radical graft copolymerization	[89]
HPC	7.0%	divinyl sulfone	chemical cross-linking	[90]
pectin	1.0–7.0%	CaCl_2_, BaCl_2_, FeCl_2_	ionic gelation	[91–93]
PVA	7.0–12.0%	–	freeze–thawing, photo-cross-linking	[55,94–96]
starch	12.0–30.0%	epichlorohydrin	chemical cross-linking	[97–98]
acrylamide/2-aminoethyl methacrylate	8%/0.19%	*N*,*N*'-methylenebisacrylamide	photopolymerization	[99]
alginate/acrylamide	2.0%/15.0%	*N,N'*-methylenebisacrylamide, CuCl_2_, ZnCl_2_, SrCl_2_, CaCl_2_	free-radical polymerization, ionic gelation	[50]
alginate/ gum arabic	3%	CaCl_2_	ionic gelation	[100]
cellulose/poly(acrylamide- *co*-acrylic acid)	5%/2.5–12.6%	methylene-bis-acrylamide	radical polymerization	[101]
chitosan, glycol/CMC, oxydized/agar	2.0%	–	physical cross-linking	[102]
chitosan/gelatin/alginate	2%/5%/2%	glutaraldehyde	chemical cross-linking	[103]
chitosan/pectin	0.2–0.7%/1.6–5.5%	CaCl_2_	ionic gelation, physical cross-linking	[104–105]
chitosan/poly (*N*-isopropylacrylamide)/ methacrylic anhydride	1.0%/2.0–4.0%/0.5%	–	UV photo-cross-linking	[106]
chitosan/PVA	1.0–10.0%	tetraethyl orthosilicate	chemical cross-linking	[107]
chitosan-poly(methacrylic acid)-poly(*N*-isopropyl- acrylamide)	0.3%	*N,N'*-methylenebisacrylamide	free-radical copolymerization	[57,108]
CMC/methyl cellulose	1%	ammonium persulfate and *N,N,N′,N*′-tetramethylethylene- diamine (initiators)	radical polymerization	[109]
CMC/xylan	25:75/50:50/75:25 mol %	ethylene glycol diglycidyl ether	radical copolymerization	[110]
collagen/hyaluronic acid	1.5%	horseradish peroxidase, H_2_O_2_	enzymatic cross-linking	[111]
collagen/γ-PGA	1.6%/0.4%	–	physical cross-linking	[112]
gelatin/chitosan	9.5%/0.5%	horseradish peroxidase, H_2_O_2_	enzymatic cross-linking	[41]
HEC/PVA	1.0%/3.0%	borax	chemical cross-linking	[113]
HEC/HPMC	2.5–7.5%/1.0–3.0%	–	temperature-oriented cross-linking	[114]
methoxyl pectin/gelatin/CMC	1.0%	glutaraldehyde, CaCl_2_	ionic gelation chemical cross-linking	[115]
pluronic F-127/*N*,*N*,*N*-trimethyl chitosan/polyethylene glycolated hyaluronic acid	13.5%/0.08%/0.08%	–	physical cross-linking	[116]
polyacrylamide/chitosan	5–15%/0.5%	*N,N'*-methylenebisacrylamide	UV photo-cross-linking	[117]
polyacrylic acid/cellulose	30%	*N,N'*-methylenebisacrylamide and *N*-hydroxysuccinimide	chemical cross-linking	[53]
PVA/alginate	12%/0.6%	CaCl_2_	ionic gelation	[62]
PVA/chitosan/starch	5.0–9.0%/1.0–10.0%/5.0–15.0%	glutaraldehyde	chemical cross-linking	[118]
PVA/starch	5.0%/3.5%	glutaraldehyde	chemical cross-linking	[119] [120]
SBMA/NAGA/CBMAX	58%	–	one pot radical polymerization	[121]

^a^Abbreviations: CMC = carboxymethyl cellulose; HEC = hydroxyethyl cellulose; HPC = hydroxypropyl cellulose; PVA = polyvinyl alcohol; PGA = polyglutamic acid; SBMA = polymerized sulfobetaine methacrylate; NAGA = *N*-(2-amino-2-oxyethyl)acrylamide; CBMAX = 1-carboxy-*N*-methyl-*N*-di(2-methacryloyloxyethyl)methanaminium inner salt.

Chitosan is one of the most versatile polymers used to prepare hydrogels. It can be combined with gelatin [122], pectin [123], hyaluronic acid [124], glucan [125], or PVA [107]. Chitosan can also be used as quaternized chitosan-g-polyaniline [126] and chitosan hydrochloride [127]. Nanofibrillated and cationized chitin, which is the precursor of chitosan, was used in the preparation of xanthan gum hydrogels [128].

Methods involving polymer mixtures generate thermo-sensitive hydrogels, such as chitosan/β-glycerophosphate/collagen-based hydrogels [129]. In another example, hydrogels comprised of gelatin microspheres, glutaraldehyde – as a cross-linker, added to Pluronic F127 and F68 can vary from sol to gel phases by changing both temperature and polymer concentration [130].

Chitosan-based hydrogels have aroused a lot of interest in the health sciences field due to their biocompatibility, biodegradability, and low cytotoxicity [131]. This makes them attractive as a promising material for topical administration of biomolecules such as nucleic acids and proteins [48,132].

Different techniques have been tested for the preparation of chitosan hydrogels, including ionotropic gelation [76], emulsion polymerization [108], and copolymerization [57,133]. Chitosan can be cross-linked with several organic and inorganic compounds due to its amine (NH_2_) and hydroxyl (OH) functional groups [77], as shown in Figure 1. The stability of hydrogels is maintained by cross-links that, in aqueous media, prevent the dissolution of polymer chains [134].

Another polymer widely used in the preparation of hydrogels is cellulose. Cellulose hydrogels have become attractive due to their high biocompatibility and long circulating time in the blood, as well as low cytotoxicity [73]. They can be prepared by various methods, including phase inversion [73], copolymerization [110–111], and double chemical cross-linking reaction [51].

Cellulose derivatives, such as cellulose ether [90], carboxymethyl cellulose (CMC) [82], hydroxyethyl cellulose (HEC) [113], or cellulose nanofibers [135] yield valuable hydrogels. Keratin hydrogels are interesting for the properties of keratin, as they have intrinsic biocompatibility, therapeutic activity, and favorable physical properties [136]. They can be formed by preparing an aqueous solution of keratin at concentrations of 8 or 20%, for example [137]. With the "disulfide shuffling" method, phosphate-buffered saline (PBS) is used to dissolve 15% lyophilized keratin and 1.5% cysteine. This solution requires vigorous stirring followed by rest to form the gel [138]. Keratin concentrations varied between 10, 15, and 20% in water, and vigorous agitation was used for dissolution [136]. Then, to promote the formation of disulfide bonds, a 5% solution of H_2_O_2_ is added. For gel formation, mixtures should be incubated overnight at 37 °C.

Collagen-based hydrogels can be developed by using different sources of collagen. Both plant and animal protein sources can provide raw materials needed for adequate collagen production, which includes rat tail tendons [84,86,139], goat tendons [87], swine skin [88], and gelatin [87]. The collagen concentration may vary in the resulting hydrogels (e.g., from 1.5% [84] to 30% [85]).

Polyvinyl alcohol hydrogels can be obtained in several ways, which include dissolving PVA in water at 90 °C with the addition of PEG-200, followed by freezing and thawing cycles. For the formation of hydrogel films, drying for four days at room temperature and storage in a desiccator is required [94]. In the method described by Gao et al. (2018), PVA 15% w/v is added into water and stirred at 95 °C until complete dissolution [55]. The freeze/thaw cycle was carried out at −20 °C and room temperature and by electronic beam irradiation.

Polyvinyl alcohol hydrogels can be used for wound healing. Suhaeri et al. (2018) manufactured an adhesive hydrogel with PVA and a derivative of human pulmonary fibroblasts, in addition to ciprofloxacin, for treating infected wounds [95]. For the preparation of the human pulmonary fibroblast derivative, the cells were cultured for seven days in a modified Eagle’s medium and then decellularized by washing several times with PBS. Then a 7% aqueous PVA solution was added to the pulmonary fibroblast derivative. For the cross-linking of the solution, the freeze/thaw method was performed, followed by ultrasound for 5 minutes at 25 °C [95]. Xu et al. (2019) dissolved PVA in water at 90 °C, with separate addition of sodium alginate and glucomannan polysaccharide [140]. The freeze/thaw process was repeated three times. The cross-linking was done with saturated solutions of Ca(OH)_2_ (pH 10.2), CaCl_2_ (pH 7.2), or CaCO_3_ (pH 8.1) for three days at room temperature, followed by washing with water and drying at 60 °C [140].

In recent years, ultrasmall nanogels have emerged as an innovative drug delivery system, mainly due to their ability to deeply penetrate biological barriers and deliver therapeutic agents with high precision and efficiency [141–143]. Among the ultrasmall systems, cyclodextrin-based nanogels have attracted considerable attention. Cyclodextrins are oligosaccharides with a hydrophobic core and a hydrophilic external surface, facilitating the encapsulation of poorly water-soluble drugs and allowing the controlled release of the encapsulated compound [142,144]. These innovations represent a significant advance in the development of multifunctional and biocompatible nanogels, suitable for applications in oncology, gene therapy, and precision medicine [145–147].

### Nanogel formulations

Nanogels can be prepared via different methods by using monomers [148] or polymers [149] as raw materials, which can be both of natural [18,150] and synthetic origin [151–152]. Depending on the composition and preparation method, nanogels can be designed to act as drug carriers to deliver hydrophobic [153] and hydrophilic [154] molecules as well as biomolecules, including proteins [155] and nucleic acids [156]. These nanocarriers can be obtained from biodegradable and biocompatible materials [157], showing singular properties, such as stimuli-responsiveness [158] and surface chemical functionalization [159], which helps to achieve high drug levels at the target site.

Several strategies have been developed to obtain nanoscale or micrometric gels instead of macroscopic networks formed by a simple gelation process [20,160–161]. In emulsion polymerization, for example, the monomer (or polymer) is emulsified in water with a suitable surfactant, and a water-soluble initiator is added to induce polymerization. After polymerization has reached the desired level, the reaction is stopped by adding a radical inhibitor [57,108,162]. Thus, the goal depends on the strategies to avoid the formation of long-range networks, which are related to the assembly of the hydrogels.

Controlling the reaction conditions is one of the strategies to obtain nanogels by cross-linking polymerization. By reducing the amount of monomers and cross-linking agents or by stopping polymerization before forming a continuous gel, the formation of small branched polymers at the nanoscale is favored over microgels [163]. This occurs because the growing chains are further apart, which makes bonds between different chains (intermolecular) more difficult and favors bonds within the same chain (intramolecular) [44,57,148].

The process of nanogel particle formation may involve physical methods [164] or chemical reactions [165]. For example, nanogels can be obtained through electrostatic interactions generated by the ionization technique with electrospray [166]. Ionizing radiation can also be used for polymer cross-linking by the formation of radical groups in the polymer chains [161]. On the other hand, chemical reactions are usually performed by applying cross-linking agents [167], such as glutaraldehyde [119,129], CaCl_2_ [74], or *N*,*N*-methylenebisacrylamide [168].

The heterogeneous polymerization process, which is carried out in a confined nano-/microscale space, is another efficient strategy for the formation of nanogels. In this case, the size of the gels is limited by confining the cross-linking to intraparticle rather than interparticle cross-linking [43–44].

### Topical and transdermal drug delivery

Topical drug delivery is a challenging issue, notably due to the barrier function of the skin, which is mainly exerted by its outermost layer, the stratum corneum (SC) [169–170]. The skin barrier is based on the specific content and composition of the SC and, in particular, on the exceptional structural organization of the intercellular lipid matrix of the SC [171–173]. The poor cutaneous permeability leads to low efficacy [170] and low selectivity [174] of drugs on the skin. Nonetheless, the development of topical delivery systems, such as nanostructured systems, has been qualified as the most helpful strategy to minimize possible side effects related to systemic drug administration. This is because, with current technologies, it is possible to perform cutaneous drug release both in a controlled and site-specific manner.

The permeability of drugs through the skin depends on the partition coefficient, the diffusion coefficient, and the path to be taken by the molecule, mainly through SC [174–176]. Overall, it is possible to improve drug penetration through the skin by using three different approaches: (i) increasing the drug saturation in the vehicle [177], (ii) elevating the drug solubility in the SC [170], and/or (iii) enhancing the drug diffusion through the SC [169,178]. The first approach can be carried out by simply increasing the drug concentration in the vehicle or by changing the formulation to eventually decrease the drug solubility (i.e., by changing the thermodynamic activity of the system). Thus, the physicochemical properties related to the first approach involve only the interactions between drugs and vehicles. On the other hand, changing the drug solubility and/or its diffusion through SC is much more complex and comprises multiple interactions among drug/vehicle, drug/skin, and skin/vehicle [169,176–177].

Besides the drug/vehicle interaction, skin permeability can be changed by the effect of vehicles on the SC [170,174,178]. Many solvents, including dimethyl sulfoxide (DMSO) [175], glycols [179], and fatty acids [180] act as penetration enhancers (i.e., they promote the disruption of the SC intercellular matrix [175] and/or extraction of the SC lipids [180]). Moreover, some penetration enhancers may operate by more than one permeation mechanism, which involves modifying either the diffusion of the drug in the SC or the solubility of the drug in the vehicle [169–170,179]. Hence, these are the basic principles for designing a new topical DDS, and all of them should be addressed to reach the goal, whether it is a nanostructured system or not.

### Hydrogel dermal applications

When obtained from natural polymers, hydrogels are efficient in tissue regeneration due to their environment, which allows the modulation of cellular behavior [181–183]. The three-dimensional structure, along with cell permeability and mechanical stability, also qualifies hydrogels as useful drug carriers. Hydrogels have been used for dermal applications due to their mechanical strength [53], high drug upload [40], controlled drug release [100], and site-specific drug delivery [184] for both topical [48] and transdermal administration [116].

Some examples of the dermal application of hydrogels as drug carriers include cutaneous wound healing [40–42] and autoimmune skin disease treatment, such as psoriasis [185–186], (enabling photoprotection [187]), and atopic dermatitis [62,188]. Hydrogels have also been used for site-specific delivery of antitumor agents in cutaneous tumor cells [189–190] as shown in Table 2.

**Table 2:** Nanogels and hydrogels for dermal applications.

DDS^a^	Properties	Model drug	Applications and outcomes	Ref.

bio-inspired alginate/gum arabic hydrogel	controlled drug release, tunable micro/nanostructures, enhanced skin adhesion	mitsugumin 53	chronic wound healing	[100]
carbon nanotube-loaded hydrogel	photothermal antimicrobial activity, skin adhesion	doxycycline	regeneration of infected skin	[41]
cellulose-poly (acrylic acid) hybrid hydrogel	pH responsiveness (swelling and drug release), high mechanical stability	amoxicillin	wound dressing	[53]
chitosan-based hydrogel	high biocompatibility and biodegradability, enhanced skin penetration	DNAzymes	prevention of enzymatic degradation	[48]
chitosan-based nanogels	pH responsiveness, site-specific drug targeting, penetration enhancer embedding	capecitabine	pH-responsive drug release mimicking the skin cancer microenvironment	[37]
chitosan/hyaluran nanogels	increase of the skin permeability	methotrexate and 5-aminole-vulinic acid	combined chemo-photodynamic therapy for psoriasis	[191]
drug-loaded coatable polyvinyl alcohol/alginate hydrogel	low cytotoxicity, anti-inflammatory effect	prednisolone	atopic dermatitis treatment	[62]
ethosomal-loaded nanogel	low particle size (≈125 nm), enhanced skin penetration, combined drug delivery	curcumin and sulforaphane	therapy against skin cancer	[192]
gellan gum nanogel incorporated into solid hydrogel film	thermo-responsiveness, penetration enhancer embedding, prolonged drug release	diclofenac, sodium	transdermal drug delivery	[32]
guanosine quartet hydrogels	high flexibility and biocompatibility, porous three-dimensional network	recombinant human-sourced collagen	wound healing and skin regeneration	[42]
HEC-based hydrogel containing deformable liposomes	low particle size (≈110 nm), sustained transdermal delivery, low dermal irritation and toxicity	methotrexate	rheumatoid arthritis treatment	[193]
HEC-based hydrogel containing ascosomes	high encapsulation efficiency (>90%), good safety profile, increased transdermal permeability	khellin	Minimize skin irritation	[194]
HPMC-based nanogel	high drug load, enhanced skin deposition, combined drug delivery	quercetin and TiO_2_	chemoprevention of skin cancer	[195]
nanocapsule/nanoemulsion- loaded chitosan hydrogel	controlled drug release, skin adhesion	phenytoin	wound healing, low risk of systemic absorption	[40]
nanostructured lipid carrier-based hydrogel	prolonged drug release, high drug deposition, high stability	mometasone furoate	psoriasis treatment	[186]
PLGA-chitosan double-walled biodegradable nanogels	high drug load, low cytotoxicity and biocompatibility, sustained drug release	5-fluorouracil	transdermal drug delivery against skin cancer	[196]
Pluronic F-127 hydrogel	pH and temperature responsiveness, sustained drug release	gallic acid	textile-based transdermal therapy	[116]
Pluronic F127-based hydrogel	low particle size (≈60 nm), fewer side-effects and a wide spectrum of action	prostaglandin derivative (15d-PGJ2)	impairment of atopic dermatitis progression	[188]
poly(*N*-isopropylacrylamide) nanogel	dual pH and temperature responsiveness, enhanced drug release into epidermis	naproxen	treatment of inflammatory skin diseases	[151]
poly(lactic-*co*-glycolic acid) nanoparticles-loaded hydrogel	low particle size (50 nm), sustained drug release, enhanced skin penetration	curcumin	psoriasis treatment	[185]
poly-*N*-vinylcaprolactam multilayered nanogel film	thermo-responsiveness (swelling and thickness), high drug load	diclofenac, sodium	sustained drug permeation through the skin, topical delivery of multiple drugs	[197]
transfersome-embedded oligopeptide hydrogel	paintable patch administration, prolonged skin retention, deformability and enhanced skin permeation	paclitaxel	topical chemotherapy of melanoma combined with systemic therapy	[189]

^a^Abbreviations: HEC = hydroxyethyl cellulose; HPMC = hydroxypropyl methylcellulose; PLGA = poly(lactic-*co*-glycolic) acid.

Chitosan hydrogels containing nanoencapsulated phenytoin showed drug release improvement, skin adherence, and increased content of both collagen fibers and fibroblasts in the wound tissue during the healing process in rats [40]. Liang et al., (2019) developed hydrogels composed of gelatin-grafted dopamine/chitosan/polydopamine-coated carbon nanotubes through the oxidative coupling of catechol groups using a hydrogen peroxide/horseradish peroxidase (H_2_O_2_/HRP) catalytic system [41]. The addition of the antibiotic doxycycline provided the hydrogels with antimicrobial activity to treat infected full-thickness defect wounds.

Flexible films composed of guanosine quartet hydrogels loaded with recombinant human-sourced collagen can be wrapped onto the skin surface. These films can supply collagen deposition for the wound by recruiting macrophages and fibroblasts and eventually inducing their proliferation and migration [42]. In the study by Zhao et al. (2017), the authors developed an innovative hydrogel that can be directly injected into the injured skin. The benefits associated with wound healing stand out: self-regeneration potential, antibacterial activity, neutralization of free radicals, high adhesion to the skin, and biocompatibility. These characteristics make the system a promising multifunctional option for treating skin wounds [126].

Topical medicines are the first-choice therapy for the management of chronic autoimmune diseases, such as psoriasis and atopic dermatitis. Skin thickening, scaling, and epidermal alterations impose many obstacles for topical therapy. Nanoparticle-loaded hydrogels have proven to be promising carrier systems to deliver mometasone furoate [186], clobetasol propionate [198], and curcumin [185] for psoriasis therapy. For treating atopic dermatitis, PVA/alginate hydrogel loaded with prednisolone showed effective suppressions of ear edema, pruritus, high IgE levels, epidermal swelling, and mast cell infiltration in Balb/c mouse model [62].

Temperature- and pH-responsive hydrogels loaded with gallic acid were developed using a combination of biocompatible polymers: Pluronic F-127, *N*,*N*,*N*-trimethyl chitosan, and polyethylene glycolated hyaluronic acid. The system was designed to be incorporated into therapeutic fabrics for transdermal application, making it a potentially valuable tool for treating conditions such as atopic dermatitis. The release mechanism of gallic acid followed a first-order kinetic model, in which the release rate decreases as the amount of gallic acid remaining in the gel diminishes [116].

### Nanogels as dermal drug carriers

**Figure 2:**
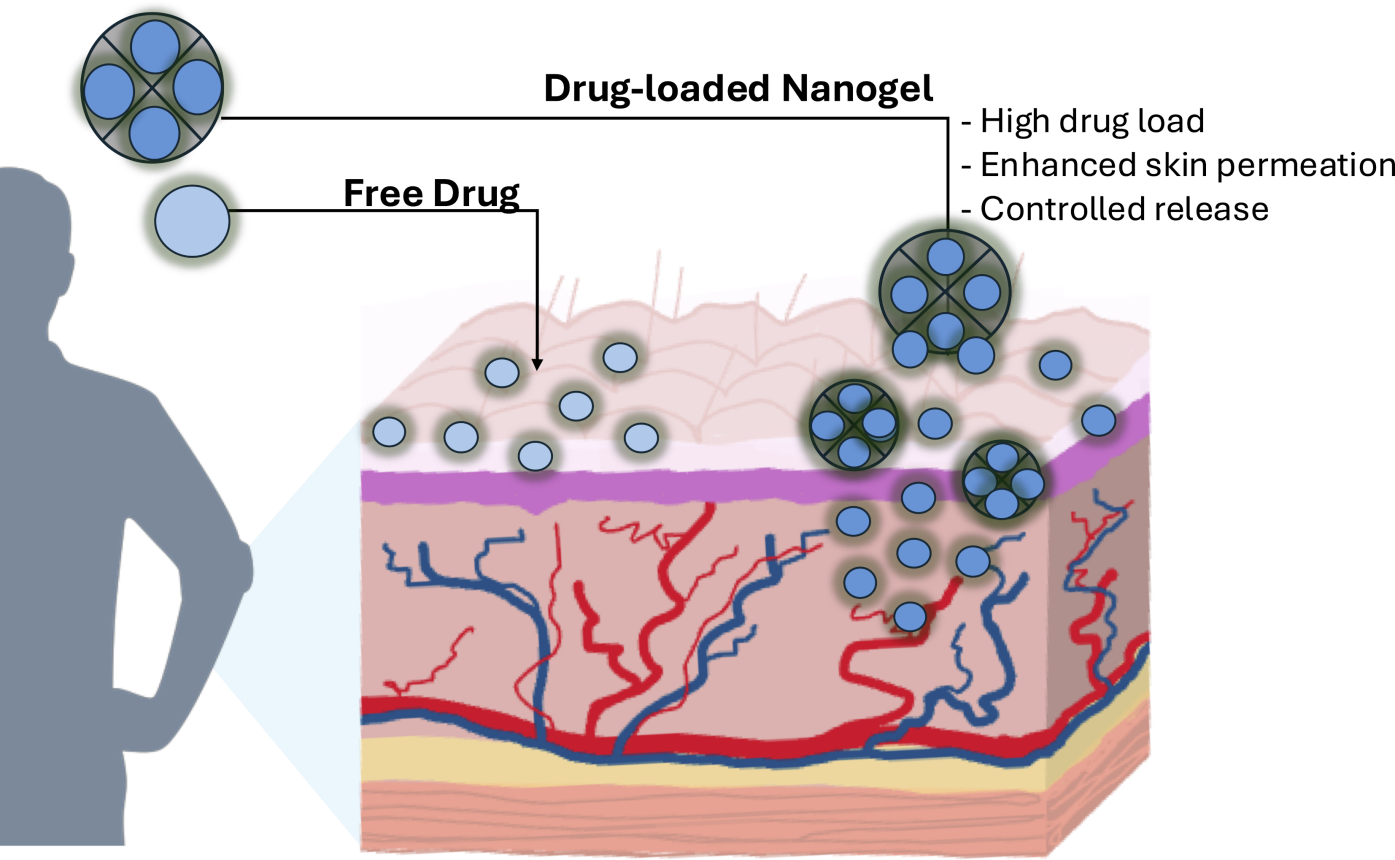
Nanogels as dermal drug delivery systems. Systems developed through the union between a polymer and a drug provide benefits by producing formulations that present increased encapsulation, increased delivery, and controlled drug release.

Recent advances have demonstrated the successful use of nanogels as drug carriers. Some benefits of nanogel applications as dermal DDSs are shown in Figure 2. It includes increased drug encapsulation [195], penetration enhancer embedding [157], stimuli responsiveness, as well as controlled drug release [37]. Due to their unique characteristics, nanogels have also shown promise as site-specific DDSs. Changes in environmental conditions, such as pH [151,199], temperature [197,200], radiation [201], reduction potential [202], or concentration of specific compounds, including glucose [203] and reactive oxygen species [204], may result in the modification of particle conformation, leading to the release of the transported drug. Some nanogels can simultaneously respond to two [205–206] or more of the aforementioned stimuli [207].

Nanogels have been explored to deliver nonsteroidal anti-inflammatory drugs into the skin. Avoiding systemic toxicity is the main reason to promote the topical and transdermal release of these drugs. Thermo-responsive nanogels consisting of multiple layers of poly(*N*-vinyl caprolactam) were developed to carry sodium diclofenac to the skin. Regarding thermo-responsiveness, the cumulative amount of diclofenac transported at 32 °C after 24 h was 12 times higher than at 22 °C [197]. In another effort, thermo-responsive nanogels based on gellan gum showed a 6-fold increase in the transport of diclofenac to the skin surface when compared with commercial formulations [32]. Nanogels synthesized from a cross-linking copolymer of poly(itaconic anhydride-*co*-3,9-divinyl-2,4,8,10-tetraoxaspiro[5.5]undecane) with 1,12-dodecanediol demonstrated dual pH and temperature responsiveness and high efficiency to release diclofenac, showing potential as dermal nanocarriers by mimicking a biological environment [208].

Naproxen, another nonsteroidal anti-inflammatory drug, has been incorporated into poly(*N*-isopropylacrylamide) nanogels for release into the skin. The nanogels showed responsiveness to pH and temperature variation. In an ex vivo test performed with rat skin, the nanogel activated by sodium carbonate revealed a significantly higher release of naproxen into the epidermis, with a 2.8-fold increase in the steady flow of the drug. A 50% reduction in the activity of the COX-2 enzyme and reduction of rat paw edema were also observed by comparing the base-activated nanogel with the nonactivated nanogel in tests confirmed by immunofixation, which corroborated the in vivo/ex vivo data [151].

Nanogels for the transdermal release of methotrexate (MTX), an antimetabolite used to treat rheumatoid arthritis, were obtained by Sadarani et al. (2019). First, methotrexate was encapsulated in deformable liposomes followed by incorporation in the hydroxyethylcellulose gel. The nanogel-MTX presented a small particle size of 110 ± 20 nm and a drug encapsulation rate of 42 ± 1.9%. In dermal toxicity studies, the nanogel-MTX formulation showed no signs of irritation or toxicity, while in the biodistribution study, the nanogel-MTX displayed sustained systemic release up to 48 hours with low accumulation in organs such as liver, kidney, and intestine. A therapeutic efficacy test was performed with a collagen-induced arthritis model. The methotrexate nanogel showed significant improvement in the hind paw edema, reduced arthritic score, reduction in joint damage (histological and radiological), and attenuation of serum cytokines levels, such as TNF-alpha and IL-6. The optimized nanogel-MTX combines the characteristics of biocompatibility, sustained systemic release, safety, and efficacy in the treatment of rheumatoid arthritis in a single system [193].

### Nanogels and hydrogels applied to skin cancer therapy

Therapy against skin cancer has been a major focus for the application of hydrogels and nanogels (Table 2), which aim to optimize site-specific drug targeting in the tumor cells [192,195,209]. Skin cancer arises through the abnormal and uncontrolled growth of cells forming skin tissue. The skin cells are arranged in layers and, according to the affected layers, different types of cancer can be discerned. Skin cancers are divided into three main types, which include squamous cell carcinoma, basal cell carcinoma, and melanoma. The most common are basal and squamous cell carcinomas. More rare and lethal than carcinomas, melanoma is the most aggressive type of skin cancer [210–212].

Polymeric nanogels [192], as well as stimulus-responsive nanogels [37,168], have proven to be effective in increasing the targeted delivery of antineoplastic drugs to fight skin cancer. Skin tumors have a microenvironment characterized by an anionic charge and a slightly acidic pH [213]. This microenvironment of skin tumors has been used to modulate site-specific drug delivery, which leads to an increase in the effectiveness of chemotherapy and also a reduction of the cytotoxicity of antineoplastic drugs [211–212]. The pH-responsive nanogel can be designed with cross-linking bonds that break when exposed to a lower pH range [214], resulting in the release of the drug at the target site. This pH-responsive behavior culminates in two favorable outcomes: decrease of side effects by reducing drug contact with healthy tissues, and potentiation of the therapeutic effect of the drug due to its higher availability at the site of action [37,215]. The pH difference observed in the intracellular environment (pH ≈ 4.8) when compared with the epidermal extracellular matrix (pH ≈ 5.5) is another characteristic evaluated to increase the efficiency of tumor-targeted drug delivery [157,212–213].

Recent advances have shown the development of polymeric nanogels for targeted delivery of antimetabolite agents in the treatment of skin cancer [168]. Antimetabolite agents are antineoplastic drugs that act by inhibiting cell division by blocking DNA and, to a lesser extent, RNA synthesis. Among the antineoplastic agents, 5-fluorouracil (5-FU) is the classic and most widely tested drug to fight skin cancer. 5-FU is a chemotherapeutic agent analogous to pyrimidine. The metabolism of 5-FU blocks the methylation reaction of deoxyuridine acid to thymidyl acid, interfering with DNA synthesis, and subsequently inhibiting the formation of RNA. The effects of reduction on DNA and RNA syntheses occur mostly in cells that proliferate more rapidly and therefore capture more 5-FU [216–217].

Among modern antimetabolite agents, capecitabine is the first-choice antineoplastic drug in contrast to 5-FU. Capecitabine is a molecule derived from fluoropyrimidine carbamate, a tumor-activated and tumor-selective cytotoxic agent. Therefore, capecitabine exerts anti-tumor action after the conversion of its molecule into 5-FU in the tumor itself. The formation of 5-FU preferably occurs by an angiogenic factor associated with the tumor, called thymidine phosphorylase (dTfdPase), thus minimizing the exposure of healthy tissues to 5-FU [216].

Biodegradable polymers are widely used in nanotechnology to develop different systems due to their advantages, such as greater absorption [218–219] and high safety [209]. Nanogels loaded with 5-FU [196,209] and capecitabine [37], when topically applied, can be an interesting strategy to improve chemotherapeutic efficiency in the treatment of skin cancer [168]. Double-walled biodegradable nanogels composed of PLGA–chitosan coated with eucalyptus oil efficiently encapsulated 5-FU [196]. The 5-FU was encapsulated in the PLGA core using the solvent evaporation technique. Then, the nanoparticle was coated with cationic chitosan, aiming to promote ionic interactions with the anionic cell membrane of the tumor. Finally, eucalyptus oil (1%) was added to the surface of the nanoparticle to favor the penetration of the nanogels into the SC). Both in vitro and ex vivo results showed higher cutaneous penetration of 5-FU performed by double-walled PLGA–chitosan nanogels coated with eucalyptus oil, demonstrating the favorable potential of nanogels in skin cancer therapy [196].

In another study, chitosan nanogels containing capecitabine promoted site-specific drug targeting directed by an ionic attraction mechanism and triggered by pH variation [37]. In this case, the drug encapsulated in chitosan was gelled using Pluronic F-127 and amended with Transcutol^®^ as a skin penetration enhancer. The cationic charge of the particles, combined with drug release in a slightly acidic environment, promoted an increase in drug permeation (ex vivo), as well as an augment in capecitabine toxicity against cancer cells in a HaCaT cell line MTT assay. This pH-sensitive behavior, together with the ionic attraction mechanism, promotes the targeted delivery of capecitabine into the tumor. The ionic attraction between the cationic surface of the particles and the anionic cell membrane of the tumor facilitates the acidic degradation of the chitosan matrix. Thus, degradation of the nanogel triggered by pH leads to the site-specific release of capecitabine in the tumor, showing a cytotoxic effect on tumor cells by what is known as enhanced permeation and retention mechanism (EPR). The EPR refers to the passive targeting technique that occurs in virtue of the vascular organization of the tumor with leakage permeable to blood flow due to disordered endothelial cell layers. This cellular organization of the vasculature with increased permeability allows increased uptake of nanoparticles by endocytosis and, consequently, increased cytotoxic effect of antineoplastic drugs. Thus, with the degradation of the nanogel (which is triggered by pH), capecitabine is released in a site-specific manner in the tumor, showing a cytotoxic effect on tumor cells [16,37].

Ethosomes are phospholipid nanovesicles containing a fraction of ethanol used for dermal and transdermal release of molecules [220–221]. Ethosomal nanogels containing sulforaphane, a potent natural antioxidant [222], showed a significant anti-cancer effect (*p* < 0.05) in murine tumor cell type B16-F10, proven to be an attractive strategy for skin cancer therapy [192].

A hydrogel system based on oligopeptides and embedded transfersomes was developed to enhance the transdermal delivery of paclitaxel, an antineoplastic agent, as a strategy for noninvasive topical chemotherapy in melanoma treatment. The transfersomes were prepared using phospholipids and surfactants, such as Tween 80 and sodium deoxycholate, to modify the lipid matrix organization and increase stratum corneum fluidity, thus favoring drug permeation. In addition, a cell-penetrating peptide (R8H3) was functionalized on the transfersome surface to improve skin and tumor penetration. The oligopeptide hydrogel composed of Fmoc-Phe-Phe-Phe-Dopa served as a drug reservoir, enhancing local drug retention. The transfersome-embedded hydrogel can be painted as a patch on the skin above the melanoma, demonstrating prolonged retention time and inhibition of the tumor growth in combination with systemic chemotherapy [189].

Nanogels for topical use in skin cancer prevention were tested by Bagde et al. (2019) [195]. The obtained nanogels were incorporated with quercetin, a natural antioxidant, and titanium dioxide (TiO_2_), which acts as an inorganic sunscreen. Formulations containing 0.08% and 0.12% quercetin exhibited an encapsulation rate of 89.3 ± 1.4% and 90.4 ± 1.8%, respectively. Nanogels containing quercetin (0.12%) and TiO_2_ (5% and 15%) showed a drug release rate above 70% with a significant increase (*p* < 0.001) in the deposition of quercetin on the skin when compared with a drug suspension in 24 hours. The mean number of tumors, tumor volume, and percentage of animals with onset tumors in the group pre-treated with quercetin nanogels (0.12%) + TiO_2_ (15%) were significantly lower (*p* < 0.05) compared with the group exposed to UV radiation (control group). Besides, the nanogel containing quercetin (0.12%) + TiO_2_ (15%) significantly reduced (*p* < 0.001) the expression of COX-2, EP3, EP4, PCNA, and cyclin D1 in contrast to the group that was pre-treated with quercetin and TiO_2_ only. Thus, nanogels containing quercetin+TiO_2_ held promise for the chemoprevention of UV-induced skin photocarcinogenesis.

In recent years, the pharmaceutical industry and the consumers have shown an increasing interest in products with features indicating environmental friendliness. This applies to production, materials, and packaging. Among the trends in the development of nanogels, one is to obtain them by methods that incorporate only biodegradable polymers in the formulation, preferably from natural origin. Thus, obtaining and characterizing nanogels by simple and rapid methods using cellulose (or carboxymethyl cellulose), lignin, or both [223–225] has been explored and exhibited biological activity. However, avoiding the use of harmful solvents and utilizing methods that enable large production of the nanogel for an eventual release of a commercial product can prove to be a challenge.

On another note, the production of gels or nanogels applied in medicine by 3D bioprinting technique has been an important tool for the treatment of some pathophysiological processes in recent years, such as tissue regeneration or tumor models in vivo. For example, 3D-printed scaffolds have been employed and shown to be effective in bone regeneration [226] and in promoting the restoration of craniofacial cartilage defects [227]. Also, in in vivo breast cancer models, doxorubicin-loaded cellulose nanocrystals poly(ε-caprolactone-*co*-lactide)-*b*-poly(ethyleneglycol)-*b*-poly(ε-caprolactone-*co*-lactide) [217] or doxorubicin-loaded polydopamine-alginate [219] showed inhibition in tumor growth.

Thus, this technique tends to present a great advance in the design methods and in the ease of obtaining them, favoring future production of tissues/organs that can be applied in the human organism.

## Conclusion

Hydrogels and nanogels, formed from various polymers, exhibit favorable characteristics for therapeutic use, especially in dermatology. These systems overcome long-standing scale-up challenges in nanocarriers, facilitating industrial manufacturing. Hydrogels, used in dermatological treatment such as wound healing films, advance the commercialization of hydrogel-based medicines. Nanogels, aiding targeted delivery, enhance the efficacy of skin cancer therapy. Their biocompatibility and ability to mimic the extracellular matrix make them attractive drug delivery systems. Dermal applications include psoriasis treatment, antimicrobial activity, wound healing, and skin tumor remission. Understanding topical and transdermal drug delivery mechanisms is crucial. Hydrogels and nanogels, proven strategies to overcome skin barriers, offer controlled and site-specific cutaneous drug release. Considerations for success include safety, efficacy, scale-up, and cost-effectiveness.

Cross-linking agents play a vital role, with biocompatible options such as TPP and genipin offering safer alternatives to cytotoxic agents. The introduction of biodegradable bonds reduces cytotoxicity. Hydrogels overcome the challenges of expansion through water-based processes, such as electrospray ionization, ensuring simplicity, safety, and economy and allowing these systems to be designed to be scalable, reproducible, and with adequate economic factors in their manufacture. Topical therapies for skin cancer, leveraging nanocarriers with active targeting, show promise. Functionalized and stimuli-responsive nanogels can accumulate in tumor cells, minimizing damage to noncarcinogenic tissues. Advancements in polymers, cross-linkers, and obtaining processes are unlocking unprecedented properties in hydrogels and nanogels, paving the way for innovative medical approaches.

## Data Availability

Data sharing is not applicable as no new data was generated or analyzed in this study.

## References

[1] Rosiak J M, Yoshii F (1999). Nucl Instrum Methods Phys Res, Sect B.

[2] Baker W O (1949). Rubber Chem Technol.

[3] Simaljakova J (1955). Cesko-Slov Farm.

[4] Buri P, Gumma A, Mirianoff A (1966). Pharm Acta Helv.

[5] Scherenzel M (1964). Pharm Acta Helv.

[6] Refojo M F (1967). J Polym Sci, Part A-1: Polym Chem.

[7] Winkler A (1970). Mycoses.

[8] Otsuka J, Amano J, Tanaka K (1968). Kaiin Dayori Nihon Kontakuto Renzu Gakkai.

[9] Hill J F (1980). Optom Vision Sci.

[10] Little S A, Bruce A S (1995). CLAO J.

[11] Ahadian S, Sadeghian R B, Salehi S, Ostrovidov S, Bae H, Ramalingam M, Khademhosseini A (2015). Bioconjugate Chem.

[12] Demirtaş T T, Irmak G, Gümüşderelioğlu M (2017). Biofabrication.

[13] Sethi S, Kaith B S, Kaur M, Sharma N, Khullar S (2019). J Biomater Sci, Polym Ed.

[14] Jha M K, Das P P, Gupta S, Chaudhary V, Gupta P, Sabu T, Bhasha S, Purnima J (2023). Complementing the circular economy with a life cycle assessment of sustainable hydrogels and their future prospects. Sustainable Hydrogels.

[15] Sharma P, Mittal H, Jindal R, Jindal D, Alhassan S M (2019). Colloids Surf, A.

[16] Karg M, Pich A, Hellweg T, Hoare T, Lyon L A, Crassous J J, Suzuki D, Gumerov R A, Schneider S, Potemkin I I (2019). Langmuir.

[17] Scotti A, Bochenek S, Brugnoni M, Fernandez-Rodriguez M A, Schulte M F, Houston J E, Gelissen A P H, Potemkin I I, Isa L, Richtering W (2019). Nat Commun.

[18] Carter P, Narasimhan B, Wang Q (2019). Int J Pharm.

[19] Argenta D F, dos Santos T C, Campos A M, Caon T, Shyam S M, Shivendu R, Nandita D (2019). Hydrogel Nanocomposite Systems: Physico-Chemical Characterization and Application for Drug-Delivery Systems. Nanocarriers for drug delivery.

[20] Ding H, Geng J, Lu Y, Zhao Y, Bai B (2020). Fuel.

[21] Han P, Geng J, Ding H, Zhang Y, Bai B (2020). Fuel.

[22] Vishnu S. K D, Ranganathan P, Rwei S-P, Pattamaprom C, Kavitha T, Sarojini P (2020). Int J Biol Macromol.

[23] Gao J, Hoshino Y, Inoue G (2020). Chem Eng J.

[24] Richa, Roy Choudhury A (2020). Carbohydr Polym.

[25] Ashrafi B, Rashidipour M, Marzban A, Soroush S, Azadpour M, Delfani S, Ramak P (2019). Carbohydr Polym.

[26] Kala S, Agarwal A, Sogan N, Naik S N, Nagpal B N, Patanjali P K, Kumar J (2019). Colloids Surf, B.

[27] Wang Y, Liu Z, Luo F, Peng H-Y, Zhang S-G, Xie R, Ju X-J, Wang W, Faraj Y, Chu L-Y (2019). J Membr Sci.

[28] Yuki Y, Uchida Y, Sawada S-i, Nakahashi-Ouchida R, Sugiura K, Mori H, Yamanoue T, Machita T, Honma A, Kurokawa S (2021). Mol Pharmaceutics.

[29] Xu X, Yuan S, Li J, Guo S, Yan Z (2023). Energy.

[30] Hashimoto Y, Mukai S-a, Sasaki Y, Akiyoshi K (2018). Adv Healthcare Mater.

[31] Stratakis E (2018). Int J Mol Sci.

[32] Carmona-Moran C A, Zavgorodnya O, Penman A D, Kharlampieva E, Bridges S L, Hergenrother R W, Singh J A, Wick T M (2016). Int J Pharm.

[33] Panonnummal R, Jayakumar R, Anjaneyan G, Sabitha M (2018). Int J Biol Macromol.

[34] Yao Y, Xia M, Wang H, Li G, Shen H, Ji G, Meng Q, Xie Y (2016). Eur J Pharm Sci.

[35] Picone P, Sabatino M A, Ditta L A, Amato A, San Biagio P L, Mulè F, Giacomazza D, Dispenza C, Di Carlo M (2018). J Controlled Release.

[36] Brannigan R P, Khutoryanskiy V V (2017). Colloids Surf, B.

[37] Sahu P, Kashaw S K, Sau S, Kushwah V, Jain S, Agrawal R K, Iyer A K (2019). Int J Biol Macromol.

[38] Zhang Z-Q, Kim Y-M, Song S-C (2019). ACS Appl Mater Interfaces.

[39] Maranduca M A, Hurjui L L, Branisteanu D C, Serban D N, Branisteanu D E, Dima N, Serban I L (2020). Exp Ther Med.

[40] Cardoso A M, de Oliveira E G, Coradini K, Bruinsmann F A, Aguirre T, Lorenzoni R, Barcelos R C S, Roversi K, Rossato D R, Pohlmann A R (2019). Mater Sci Eng, C.

[41] Liang Y, Zhao X, Hu T, Han Y, Guo B (2019). J Colloid Interface Sci.

[42] Xiao M, Gao L, Chandrasekaran A R, Zhao J, Tang Q, Qu Z, Wang F, Li L, Yang Y, Zhang X (2019). Mater Sci Eng: C.

[43] Oyama T, Kobayashi S, Mullen K (2014). Cross-linked polymers synthesis. Encyclopedia of Polymeric Nanomaterials.

[44] Sanson N, Rieger J (2010). Polym Chem.

[45] Patil S, Jadge D R (2008). Pharm Rev.

[46] Azeredo H M C, Waldron K W (2016). Trends Food Sci Technol.

[47] Tillet G, Boutevin B, Ameduri B (2011). Prog Polym Sci.

[48] Eicher A-C, Dobler D, Kiselmann C, Schmidts T, Runkel F (2019). Int J Pharm.

[49] Martínez-Martínez M, Rodríguez-Berna G, Bermejo M, Gonzalez-Alvarez I, Gonzalez-Alvarez M, Merino V (2019). Eur J Pharm Biopharm.

[50] Zhou Q, Kang H, Bielec M, Wu X, Cheng Q, Wei W, Dai H (2018). Carbohydr Polym.

[51] Ye D, Chang C, Zhang L (2019). Biomacromolecules.

[52] Yu F, Yang P, Yang Z, Zhang X, Ma J (2021). Chem Eng J.

[53] Chuah C, Wang J, Tavakoli J, Tang Y (2018). Polymers (Basel, Switz).

[54] Wang T, Qu G, Wang C, Cheng Y, Shang J, Zheng J, Feng Z, Chen Q, He N (2019). Langmuir.

[55] Gao D, Zhou X, Gao Z, Shi X, Wang Z, Wang Y, Zhang P (2018). J Pharm Sci.

[56] Furuike T, Komoto D, Hashimoto H, Tamura H (2017). Int J Biol Macromol.

[57] Rasib S Z M, Akil H M, Yahya A S (2016). Procedia Chem.

[58] Miloudi L, Bonnier F, Bertrand D, Byrne H J, Perse X, Chourpa I, Munnier E (2017). Anal Bioanal Chem.

[59] Xu W, Huang L, Jin W, Ge P, Shah B R, Zhu D, Jing J (2019). Int J Biol Macromol.

[60] Akhtar M F, Ranjha N M, Hanif M (2015). Daru, J Pharm Sci.

[61] Cong Z, Shi Y, Wang Y, Wang Y, Niu J, Chen N, Xue H (2018). Int J Biol Macromol.

[62] Lee H R, Kim T H, Oh S H, Lee J H (2018). J Biomater Sci, Polym Ed.

[63] Bertsch P, Diba M, Mooney D J, Leeuwenburgh S C G (2023). Chem Rev.

[64] Cao J, Huang D, Peppas N A (2020). Adv Drug Delivery Rev.

[65] Beach M A, Nayanathara U, Gao Y, Zhang C, Xiong Y, Wang Y, Such G K (2024). Chem Rev.

[66] Mitchell M J, Billingsley M M, Haley R M, Wechsler M E, Peppas N A, Langer R (2021). Nat Rev Drug Discovery.

[67] Tanasa E, Zaharia C, Radu I-C, Surdu V-A, Vasile B S, Damian C-M, Andronescu E (2019). Nanomaterials.

[68] Fan Y, Wu W, Lei Y, Gaucher C, Pei S, Zhang J, Xia X (2019). Mar Drugs.

[69] Gao B, Chen L, Zhao Y, Yan X, Wang X, Zhou C, Shi Y, Xue W (2019). Eur Polym J.

[70] Sun X, Ma C, Gong W, Ma Y, Ding Y, Liu L (2020). Int J Biol Macromol.

[71] Ma W, Zhang H, Ma H, Wu C (2022). Prog Nat Sci: Mater Int.

[72] Wang T, Zheng Y, Shi Y, Zhao L (2019). Drug Delivery Transl Res.

[73] Jiang H, Tovar-Carrillo K, Kobayashi T (2016). Ultrason Sonochem.

[74] Zhang X-F, Ma X, Hou T, Guo K, Yin J, Wang Z, Shu L, He M, Yao J (2019). Angew Chem, Int Ed.

[75] Luo Y, Mills D K (2019). Gels.

[76] Ashrafi H, Azadi A (2016). Int J Biol Macromol.

[77] Eivazzadeh-Keihan R, Radinekiyan F, Maleki A, Salimi Bani M, Hajizadeh Z, Asgharnasl S (2019). Int J Biol Macromol.

[78] Rodríguez-Acosta H, Tapia- Rivera J M, Guerrero-Guzmán A, Hernández-Elizarraráz E, Hernández- Díaz J A, Garza- García J J O, Pérez- Ramírez P E, Velasco- Ramírez S F, Ramírez- Anguiano A C, Velázquez- Juárez G (2022). J Tissue Viability.

[79] Liu Y, Cai Z, Sheng L, Ma M, Xu Q, Jin Y (2019). Carbohydr Polym.

[80] Pawar V, Dhanka M, Srivastava R (2019). Colloids Surf, B.

[81] Yüksel Aslıer N G, Tağaç A A, Durankaya S M, Çalışır M, Ersoy N, Kırkım G, Yurdakoç K, Bağrıyanık H A, Yılmaz O, Sütay S (2019). Int J Pediatr Otorhinolaryngol.

[82] Ali N H, Amin M C I M, Ng S-F (2019). J Biomater Sci, Polym Ed.

[83] Enoch K, Somasundaram A A (2023). Int J Biol Macromol.

[84] McCoy M G, Seo B R, Choi S, Fischbach C (2016). Acta Biomater.

[85] Yuan X, Wei Y, Villasante A, Ng J J D, Arkonac D E, Chao P-h G, Vunjak-Novakovic G (2017). Biomaterials.

[86] Samadian H, Vaez A, Ehterami A, Salehi M, Farzamfar S, Sahrapeyma H, Norouzi P (2019). J Mater Sci: Mater Med.

[87] Goodarzi H, Jadidi K, Pourmotabed S, Sharifi E, Aghamollaei H (2019). Int J Biol Macromol.

[88] Levato R, Webb W R, Otto I A, Mensinga A, Zhang Y, van Rijen M, van Weeren R, Khan I M, Malda J (2017). Acta Biomater.

[89] Wang C, Niu H, Ma X, Hong H, Yuan Y, Liu C (2019). ACS Appl Mater Interfaces.

[90] Kato N, Gehrke S H (2004). Colloids Surf, B.

[91] Kongkaew W, Sangwan W, Lerdwijitjarud W, Sirivat A (2018). J Biomater Appl.

[92] Markov P A, Krachkovsky N S, Durnev E A, Martinson E A, Litvinets S G, Popov S V (2017). J Biomed Mater Res, Part A.

[93] Markov P A, Khramova D S, Shumikhin K V, Nikitina I R, Beloserov V S, Martinson E A, Litvinets S G, Popov S V (2019). J Biomed Mater Res, Part A.

[94] Borzenkov M, D’Alfonso L, Polissi A, Sperandeo P, Collini M, Dacarro G, Taglietti A, Chirico G, Pallavicini P (2019). Nanotechnology.

[95] Suhaeri M, Noh M H, Moon J-H, Kim I G, Oh S J, Ha S S, Lee J H, Park K (2018). Theranostics.

[96] Gao T, Jiang M, Liu X, You G, Wang W, Sun Z, Ma A, Chen J (2019). Polymers (Basel, Switz).

[97] Gholamali I, Hosseini S N, Alipour E, Yadollahi M (2019). Starch/Staerke.

[98] Namazi H, Hasani M, Yadollahi M (2019). Int J Biol Macromol.

[99] Stanton A E, Tong X, Yang F (2019). APL Bioeng.

[100] Li M, Li H, Li X, Zhu H, Xu Z, Liu L, Ma J, Zhang M (2017). ACS Appl Mater Interfaces.

[101] Ariaeenejad S, Hosseini E, Motamedi E, Moosavi-Movahedi A A, Salekdeh G H (2019). Chem Eng J.

[102] Cho I S, Ooya T (2019). Int J Biol Macromol.

[103] Sharma C, Dinda A K, Potdar P D, Chou C-F, Mishra N C (2016). Mater Sci Eng: C.

[104] Long J, Etxeberria A E, Nand A V, Bunt C R, Ray S, Seyfoddin A (2019). Mater Sci Eng: C.

[105] Torpol K, Sriwattana S, Sangsuwan J, Wiriyacharee P, Prinyawiwatkul W (2019). Int J Food Sci Technol.

[106] Wang L, Li B, Xu F, Xu Z, Wei D, Feng Y, Wang Y, Jia D, Zhou Y (2017). Carbohydr Polym.

[107] Thanyacharoen T, Chuysinuan P, Techasakul S, Nooeaid P, Ummartyotin S (2018). Int J Biol Macromol.

[108] Md Rasib S Z, Md Akil H, Khan A, Abdul Hamid Z A (2019). Int J Biol Macromol.

[109] Lin H A, Varma D M, Hom W W, Cruz M A, Nasser P R, Phelps R G, Iatridis J C, Nicoll S B (2019). J Mech Behav Biomed Mater.

[110] Kundu D, Banerjee T (2019). ACS Omega.

[111] Ying H, Zhou J, Wang M, Su D, Ma Q, Lv G, Chen J (2019). Mater Sci Eng: C.

[112] Cho S-H, Kim A, Shin W, Heo M B, Noh H J, Hong K S, Cho J-H, Lim Y T (2017). Int J Nanomed.

[113] Huang S, Su S, Gan H, Linjun W, Lin C, Danyuan X, Zhou H, Lin X, Qin Y (2019). Carbohydr Polym.

[114] Binder L, Mazál J, Petz R, Klang V, Valenta C (2019). Skin Res Technol.

[115] Jantrawut P, Bunrueangtha J, Suerthong J, Kantrong N (2019). Materials.

[116] Chatterjee S, Hui P C-l, Kan C-w, Wang W (2019). Sci Rep.

[117] Jung S, Abel J H, Starger J L, Yi H (2016). Biomacromolecules.

[118] Adeli H, Khorasani M T, Parvazinia M (2019). Int J Biol Macromol.

[119] Hassan A, Niazi M B K, Hussain A, Farrukh S, Ahmad T (2018). J Polym Environ.

[120] Batool S, Hussain Z, Niazi M B K, Liaqat U, Afzal M (2019). J Drug Delivery Sci Technol.

[121] Huangfu Y, Li S, Deng L, Zhang J, Huang P, Feng Z, Kong D, Wang W, Dong A (2021). ACS Appl Mater Interfaces.

[122] Cheng Y-H, Ko Y-C, Chang Y-F, Huang S-H, Liu C J-l (2019). Exp Eye Res.

[123] Morello G, De Iaco G, Gigli G, Polini A, Gervaso F (2023). Gels.

[124] Mohan N, Mohanan P V, Sabareeswaran A, Nair P (2017). Int J Biol Macromol.

[125] Jiang C, Wang X, Wang G, Hao C, Li X, Li T (2019). Composites, Part B.

[126] Zhao X, Wu H, Guo B, Dong R, Qiu Y, Ma P X (2017). Biomaterials.

[127] Fabiano A, Bizzarri R, Zambito Y (2017). Int J Nanomed.

[128] Kawano A, Sato K, Yamamoto K, Kadokawa J-i (2019). J Polym Environ.

[129] Shan J, Tang B, Liu L, Sun X, Shi W, Yuan T, Liang J, Fan Y, Zhang X (2019). Mater Sci Eng: C.

[130] Liu J, Chen Z, Wang J, Li R, Li T, Chang M, Yan F, Wang Y (2018). ACS Appl Mater Interfaces.

[131] Shan J, Yu Y, Liu X, Chai Y, Wang X, Wen G (2024). Heliyon.

[132] Ouyang Q-Q, Hu Z, Lin Z-P, Quan W-Y, Deng Y-F, Li S-D, Li P-W, Chen Y (2018). Int J Biol Macromol.

[133] Dong X, Wei C, Liang J, Liu T, Kong D, Lv F (2017). Colloids Surf, B.

[134] Raemdonck K, Demeester J, De Smedt S (2009). Soft Matter.

[135] Yang X, Biswas S K, Yano H, Abe K (2020). Cellulose.

[136] Wang J, Hao S, Luo T, Cheng Z, Li W, Gao F, Guo T, Gong Y, Wang B (2017). Colloids Surf, B.

[137] Tomblyn S, Pettit Kneller E L, Walker S J, Ellenburg M D, Kowalczewski C J, Van Dyke M, Burnett L, Saul J M (2016). J Biomed Mater Res, Part B.

[138] Cao Y, Yao Y, Li Y, Yang X, Cao Z, Yang G (2019). J Colloid Interface Sci.

[139] She J, Liu J, Mu Y, Lv S, Tong J, Liu L, He T, Wang J, Wei D (2025). React Funct Polym.

[140] Xu N, Yang H, Wei R, Pan S, Huang S, Xiao X, Wen H, Xue W (2019). Int J Biol Macromol.

[141] Kimura A, Jo J-i, Yoshida F, Hong Z, Tabata Y, Sumiyoshi A, Taguchi M, Aoki I (2021). Acta Biomater.

[142] Takeuchi S, Cesari A, Soma S, Suzuki Y, Casulli M A, Sato K, Mancin F, Hashimoto T, Hayashita T (2023). Chem Commun.

[143] Wang X, Peng Y, Peña J, Xing J (2021). J Colloid Interface Sci.

[144] Topuz F, Uyar T (2022). Carbohydr Polym.

[145] Machado R L, Gomes A C, Marques E F (2024). J Mol Liq.

[146] Afraz S, Ghasemzadeh H, Dargahi M (2022). Mater Today Chem.

[147] Lu P, Ruan D, Huang M, Tian M, Zhu K, Gan Z, Xiao Z (2024). Signal Transduction Targeted Ther.

[148] Theune L E, Charbaji R, Kar M, Wedepohl S, Hedtrich S, Calderón M (2019). Mater Sci Eng: C.

[149] Stickdorn J, Nuhn L (2020). Eur Polym J.

[150] Rosso A P, Martinelli M (2020). Eur Polym J.

[151] Yurdasiper A, Ertan G, Heard C M (2018). Nanomedicine (N Y, NY, U S).

[152] Peng H, Huang X, Melle A, Karperien M, Pich A (2019). J Colloid Interface Sci.

[153] Li X-M, Wu Z-Z, Zhang B, Pan Y, Meng R, Chen H-Q (2019). Food Chem.

[154] Peres L B, dos Anjos R S, Tappertzhofen L C, Feuser P E, de Araújo P H H, Landfester K, Sayer C, Muñoz-Espí R (2018). Eur Polym J.

[155] Kordalivand N, Li D, Beztsinna N, Sastre Torano J, Mastrobattista E, van Nostrum C F, Hennink W E, Vermonden T (2018). Chem Eng J.

[156] Khaled S Z, Cevenini A, Yazdi I K, Parodi A, Evangelopoulos M, Corbo C, Scaria S, Hu Y, Haddix S G, Corradetti B (2016). Biomaterials.

[157] Sahu P, Kashaw S K, Kushwah V, Sau S, Jain S, Iyer A K (2017). Bioorg Med Chem.

[158] Wang X, Luo J, He L, Cheng X, Yan G, Wang J, Tang R (2018). J Colloid Interface Sci.

[159] Mauri E, Veglianese P, Papa S, Mariani A, De Paola M, Rigamonti R, Chincarini G M F, Rimondo S, Sacchetti A, Rossi F (2017). Eur Polym J.

[160] Ashrafizadeh M, Tam K C, Javadi A, Abdollahi M, Sadeghnejad S, Bahramian A (2019). J Colloid Interface Sci.

[161] Duygu Sütekin S, Güven O (2019). Appl Radiat Isot.

[162] Simič R, Mandal J, Zhang K, Spencer N D (2021). Soft Matter.

[163] Navarro L, Theune L E, Calderón M (2020). Eur Polym J.

[164] Matusiak M, Kadlubowski S, Ulanski P (2018). Radiat Phys Chem.

[165] Liu N, Liu H, Chen H, Wang G, Teng H, Chang Y (2020). Colloids Surf, B.

[166] Simonson A W, Lawanprasert A, Goralski T D P, Keiler K C, Medina S H (2019). Nanomedicine (N Y, NY, U S).

[167] Can M, Ayyala R S, Sahiner N (2019). J Colloid Interface Sci.

[168] Zhang Q, Colazo J, Berg D, Mugo S M, Serpe M J (2017). Mol Pharmaceutics.

[169] Couto A, Fernandes R, Cordeiro M N S, Reis S S, Ribeiro R T, Pessoa A M (2014). J Controlled Release.

[170] Li B S, Cary J H, Maibach H I (2018). Arch Dermatol Res.

[171] Bäsler K, Bergmann S, Heisig M, Naegel A, Zorn-Kruppa M, Brandner J M (2016). J Controlled Release.

[172] Elias P M (2017). Exp Dermatol.

[173] Schmitt T, Neubert R H H (2018). Chem Phys Lipids.

[174] Ng K W (2018). Pharmaceutics.

[175] Marwah H, Garg T, Goyal A K, Rath G (2016). Drug Delivery.

[176] Lundborg M, Wennberg C L, Narangifard A, Lindahl E, Norlén L (2018). J Controlled Release.

[177] Chen L, Han L, Saib O, Lian G (2015). Pharm Res.

[178] Kapoor M S, GuhaSarkar S, Banerjee R (2017). Ther Delivery.

[179] Amjadi M, Mostaghaci B, Sitti M (2017). Curr Gene Ther.

[180] Gupta R, Dwadasi B S, Rai B, Mitragotri S (2019). Sci Rep.

[181] Choi W I, Hwang Y, Sahu A, Min K, Sung D, Tae G, Chang J H (2018). Biomater Sci.

[182] Li X, Cho B, Martin R, Seu M, Zhang C, Zhou Z, Choi J S, Jiang X, Chen L, Walia G (2019). Sci Transl Med.

[183] Zhao X, Lang Q, Yildirimer L, Lin Z Y, Cui W, Annabi N, Ng K W, Dokmeci M R, Ghaemmaghami A M, Khademhosseini A (2016). Adv Healthcare Mater.

[184] Mir M, Ahmed N, Permana A D, Rodgers A M, Donnelly R F, Rehman A u (2019). Pharmaceutics.

[185] Sun L, Liu Z, Wang L, Cun D, Tong H H Y, Yan R, Chen X, Wang R, Zheng Y (2017). J Controlled Release.

[186] Kaur N, Sharma K, Bedi N (2018). Pharm Nanotechnol.

[187] Batista C M, de Queiroz L A, Alves Â V F, Reis E C A, Santos F A, Castro T N, Lima B S, Araújo A A S, Godoy C A P, Severino P (2022). Heliyon.

[188] Napimoga M H, Clemente-Napimoga J T, Machabanski N M, Juliani M E A, Acras P H B C, Macedo C G, Abdalla H B, de Pinho A J, Soares A B, Sperandio M (2019). Mol Med Rep.

[189] Jiang T, Wang T, Li T, Ma Y, Shen S, He B, Mo R (2018). ACS Nano.

[190] Brianna, Anwar A, Teow S-Y, Wu Y S (2024). J Drug Delivery Sci Technol.

[191] Wang Y, Fu S, Lu Y, Lai R, Liu Z, Luo W, Xu Y (2022). Carbohydr Polym.

[192] Soni K, Mujtaba A, Akhter M H, Zafar A, Kohli K (2020). J Microencapsulation.

[193] Sadarani B, Majumdar A, Paradkar S, Mathur A, Sachdev S, Mohanty B, Chaudhari P (2019). Biomed Pharmacother.

[194] Risaliti L, Yu X, Vanti G, Bergonzi M C, Wang M, Bilia A R (2021). Int J Biol Macromol.

[195] Bagde A, Patel K, Mondal A, Kutlehria S, Chowdhury N, Gebeyehu A, Patel N, Kumar N, Singh M (2019). AAPS PharmSciTech.

[196] Sahu P, Kashaw S K, Jain S, Sau S, Iyer A K (2017). J Controlled Release.

[197] Zavgorodnya O, Carmona-Moran C A, Kozlovskaya V, Liu F, Wick T M, Kharlampieva E (2017). J Colloid Interface Sci.

[198] Kumar S, Prasad M, Rao R (2021). Mater Sci Eng: C.

[199] Zheng Y, Lv X, Xu Y, Cheng X, Wang X, Tang R (2019). Int J Biol Macromol.

[200] Mirrahimi M, Abed Z, Beik J, Shiri I, Shiralizadeh Dezfuli A, Pirhajati Mahabadi V, Kamrava S K, Ghaznavi H, Shakeri-Zadeh A (2019). Pharmacol Res.

[201] Hang C, Zou Y, Zhong Y, Zhong Z, Meng F (2017). Colloids Surf, B.

[202] Phan Q T, Patil M P, Tu T T K, Kim G-D, Lim K T (2020). React Funct Polym.

[203] Elshaarani T, Yu H, Wang L, Lin L, Wang N, ur Rahman Naveed K, Zhang L, Han Y, Fahad S, Ni Z (2020). Eur Polym J.

[204] Zhang Y, Ma C, Zhang S, Wei C, Xu Y, Lu W (2018). Mater Today Chem.

[205] Ghaeini-Hesaroeiye S, Boddohi S, Vasheghani-Farahani E (2020). Int J Biol Macromol.

[206] Qu Y, Chu B, Wei X, Lei M, Hu D, Zha R, Zhong L, Wang M, Wang F, Qian Z (2019). J Controlled Release.

[207] Huang Y, Tang Z, Peng S, Zhang J, Wang W, Wang Q, Lin W, Lin X, Zu X, Luo H (2020). React Funct Polym.

[208] Nita L E, Chiriac A P, Diaconu A, Tudorachi N, Mititelu-Tartau L (2016). Int J Pharm.

[209] Sahu P, Kashaw S K, Sau S, Kushwah V, Jain S, Agrawal R K, Iyer A K (2019). Colloids Surf, B.

[210] Barton V, Armeson K, Hampras S, Ferris L K, Visvanathan K, Rollison D, Alberg A J (2017). Arch Dermatol Res.

[211] Goyal N, Thatai P, Sapra B (2017). Ther Delivery.

[212] Collins L, Quinn A, Stasko T (2019). Dermatol Clin.

[213] Cuggino J C, Molina M, Wedepohl S, Alvarez Igarzabal C I, Calderón M, Gugliotta L M (2016). Eur Polym J.

[214] Yang G, Fu S, Yao W, Wang X, Zha Q, Tang R (2017). J Colloid Interface Sci.

[215] Xiong W, Wang W, Wang Y, Zhao Y, Chen H, Xu H, Yang X (2011). Colloids Surf, B.

[216] Collins A, Savas J, Doerfler L (2019). Dermatol Clin.

[217] Phan V H G, Murugesan M, Huong H, Le T-T, Phan T-H, Manivasagan P, Mathiyalagan R, Jang E-S, Yang D C, Li Y (2022). ACS Appl Mater Interfaces.

[218] Khalil N M, do Nascimento T C F, Casa D M, Dalmolin L F, de Mattos A C, Hoss I, Romano M A, Mainardes R M (2013). Colloids Surf, B.

[219] Wei Y, Gao L, Wang L, Shi L, Wei E, Zhou B, Zhou L, Ge B (2017). Drug Delivery.

[220] Das S K, Chakraborty S, Roy C, Rajabalaya R, Mohaimin A W, Khanam J, Nanda A, David S R (2018). Curr Drug Delivery.

[221] Pilch E, Musiał W (2018). Int J Mol Sci.

[222] Vanduchova A, Anzenbacher P, Anzenbacherova E (2019). J Med Food.

[223] Sathasivam T, Hu L, Sugiarto S, Dou Q, Zhang Z, Tan H R, Leow Y, Zhu Q, Lee C-L K, Yu H-D (2022). Chem – Asian J.

[224] Luo T, Hao Y, Wang C, Jiang W, Ji X, Yang G, Chen J, Janaswamy S, Lyu G (2022). Nanomaterials.

[225] Zhang W, Shen J, Gao P, Jiang Q, Xia W (2022). Ind Crops Prod.

[226] Miao Y, Chen Y, Luo J, Liu X, Yang Q, Shi X, Wang Y (2023). Bioact Mater.

[227] Wu G, Lu L, Ci Z, Wang Y, Shi R, Zhou G, Li S (2022). Front Bioeng Biotechnol.

